# Continuous Spinning of High‐Tough Hydrogel Fibers for Flexible Electronics by Using Regional Heterogeneous Polymerization

**DOI:** 10.1002/advs.202305226

**Published:** 2023-10-27

**Authors:** Shaoji Wu, Caihong Gong, Zichao Wang, Sijia Xu, Wen Feng, Zhiming Qiu, Yurong Yan

**Affiliations:** ^1^ School of Materials Science and Engineering South China University of Technology Guangzhou 510641 P. R. China; ^2^ Guangzhou Fiber Product Testing Institute Guangzhou 511447 P. R. China; ^3^ Key Lab of Guangdong High Property & Functional Polymer Materials Guangzhou 510640 P. R. China

**Keywords:** flexible electronics, hydrogel fiber, regional heterogeneous polymerization, self‐lubricating spinning, toughness

## Abstract

Hydrogel fibers have attracted substantial interest for application in flexible electronics due to their ionic conductivity, high specific surface area, and ease of constructing multidimensional structures. However, universal continuous spinning methods for hydrogel fibers are yet lacking. Based on the hydrophobic mold induced regional heterogeneous polymerization, a universal self‐lubricating spinning (SLS) strategy for the continuous fabrication of hydrogel fibers from monomers is developed. The universality of the SLS strategy is demonstrated by the successful spinning of 10 vinyl monomer‐based hydrogel fibers. Benefiting from the universality of the SLS strategy, the SLS strategy can be combined with pre‐gel design and post‐treatment toughening to prepare highly entangled polyacrylamide (PAM) and ionic crosslinked poly(acrylamide‐*co*‐acrylic acid)/Fe^3+^ (W‐PAMAA/Fe^3+^) hydrogel fibers, respectively. In particular, the W‐PAMAA/Fe^3+^ hydrogel fiber exhibited excellent mechanical properties (tensile stress > 4 MPa, tensile strain > 400%) even after 120 days of swelling in the pH of 3–9. Furthermore, owing to the excellent multi‐faceted performance and one‐dimensionality of W‐PAMAA/Fe^3+^ hydrogel fibers, flexible sensors with different dimensions and functions can be constructed bottom‐up, including the one‐dimensional (1D) strain sensor, two‐dimensional (2D) direction sensor, three‐dimensional (3D) pressure sensor, and underwater communication sensor to present the great potential of hydrogel fibers in flexible electronics.

## Graphical abstract

A universal self‐lubricating spinning strategy (SLS) is developed for the continuous fabrication of hydrogel fibers and its spinning mechanism is discussed. The high‐tough hydrogel fibers prepared based on the SLS strategy demonstrate the ability to construct flexible electronic devices from the bottom up.

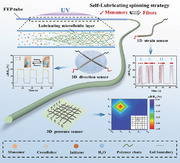

## 1 Introduction

Conductive hydrogels are attractive in recent flexible electronics applications due to their high stretchability, unique ionic conductive pathways, and similarity to biological tissues.^[^ ^1^ ^]^ Remarkably, current conductive hydrogel sensors are mainly fabricated from hydrogel‐based blocks or membranes to convert external stimuli into collectable electrical signals, which to some extent hinders their application in more diverse environments.^[^ ^2^ ^]^ In addition, the large size and isotropic homogeneous structure of hydrogel blocks or membrane‐based sensors are also unfavorable for their response and sensitivity.^[^ ^3^ ^]^ Unlike hydrogel blocks or membranes, hydrogel fibers are 1D materials with high specific surface area and ease of building 2 and 3D structures, which can miniaturize the size of hydrogels to increase the effective sensing area and meet various other sensing requirements. However, only limited methods have been developed to fabricate hydrogel fibers with restricted lengths and unsatisfactory mechanical properties. There is a great need to develop effective spinning strategies for the continuous fabrication of hydrogel fibers and to ensure their suitable mechanical properties that allow them to construct complex structures and maintain their structural integrity.

Several methods have been developed to fabricate hydrogel fibers, including electrospinning,^[^ ^4^ ^]^ draw‐spinning,^[^ ^5^ ^]^ 3D printing,^[^ ^6^ ^]^ and wet‐spinning.^[^ ^7^ ^]^ These methods mainly utilize the rapid volatilization or exchange of the solvent phase or the fast curing of the spinning solution to achieve the forming of hydrogel fibers, which require elaborative selection and modification of the solvent or spinning solution, limiting the choice of materials that can be used for hydrogel fiber fabrication. Moreover, these fast‐forming hydrogel fibers usually exhibit lower mechanical properties due to their inhomogeneous network structure. The microfluidic spinning method accomplished the continuous fabrication of hydrogel fibers by creating a mobile phase around the tube wall to isolate the hydrogel fiber from the tube wall.^[^ ^8^ ^]^ Inspired by the microfluidic approach, Zhao et al. demonstrated an efficient approach toward the continuous fabrication of an electro‐responsive hydrogel fiber by using a self‐lubricated spinning strategy. Briefly, the poly(2‐acrylamido‐2‐methylpropanesulfonic acid) (PAMPS) induced polymerization of acrylamide (AM) monomer generated a lubricating solution layer, which prevented the contact of hydrogel fibers with the tube wall to continuously fabricate electro‐reactive hydrogel fibers.^[^ ^9^ ^]^ However, the necessary introduction of PAMPS was unfavorable for the free design of hydrogel networks. In recent years, Zhu and co‐workers have innovatively combined the reactive spinning method and drafting processes to prepare a variety of hydrogel fibers with high mechanical properties.^[^ ^10^ ^]^ Unfortunately, the reactive‐draft spinning strategy utilized the low resilience of weakly crosslinked hydrogels to successfully integrate with the drafting process, which made it difficult to be compatible with existing high‐elasticity hydrogel network design strategies. Obviously, developing a universal continuous spinning strategy for preparing hydrogel fibers with a network structure that could be freely designed was a great challenge.

In this study, we developed a universal SLS strategy for the continuous spinning of high‐tough hydrogel fibers by using the hydrophobic mold induced regional heterogeneous polymerization. Ten vinyl monomers were chosen to demonstrate the universality of the SLS strategy. Additionally, the SLS strategy combined with highly entangled and ionic crosslinking network design strategies to prepare PAM hydrogel fibers with tensile stress/strain of 0.63 ± 0.42 MPa/1229.02 ± 80.62% and W‐PAMAA/Fe^3+^ hydrogel fibers with tensile stress/strain of 6.61 ± 0.38 MPa/577.97 ± 40.61%, respectively. Considering the excellent mechanical properties of the W‐PAMAA/Fe^3+^ hydrogel fiber and the lesser toughening mechanism studies, we systematically presented the toughening mechanism of W‐PAMAA/Fe^3+^ in terms of changes in the elemental components, ionic coordination, and hydrogen bonds. The W‐PAMAA/Fe^3+^ hydrogel fiber exhibited long‐term mechanical stability in an environment with a pH of 3–9 due to the synergistic effect of hydrogen bonding and ionic coordination. Moreover, we also demonstrated the feasibility of fabricating multidimensional hydrogel fiber‐based sensors by simple weaving and assembly. These hydrogel fiber‐based sensors were capable of converting mechanical stimuli such as tensile strain, tensile direction, and pressure into collectable electrical signals for human motion monitoring, pressure level/pressure position detection, and underwater communication. This work provided a universal continuous spinning strategy that was compatible with existing hydrogel design strategies for fabricating high‐tough, multifunctional hydrogel fiber‐based flexible sensors.

## 2 Results and Discussion

### 2.1 The Self‐Lubricating Phenomena and Mechanisms of Hydrogel Fibers

**Figure 1:**
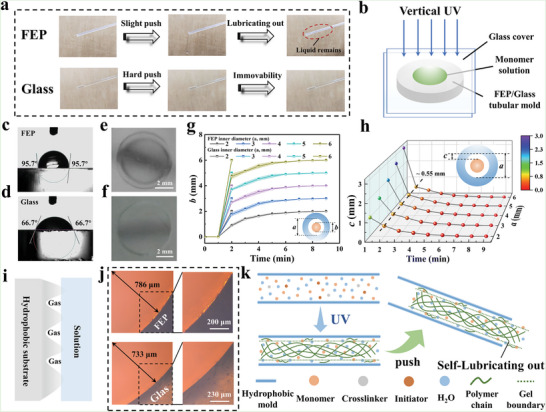
a) Differences in self‐lubrication of AM solutions polymerized in the FEP and glass tube. b) Schematic diagram of vertical UV polymerization experiment. The hydrophilicity of the c) FEP and d) glass mold. The regional heterogeneity of AM solutions polymerized in the e) FEP and f) glass mold. g,h) Size changes of hydrogel when AM solutions were polymerized in the FEP and glass mold. i) Schematic illustration of the wetting behavior of aqueous solutions on a rough hydrophobic material. j) Optical image of methyl orange stained AM solution at FEP and glass surfaces. k) The self‐lubrication model.

The fabcrication of hydrogel fibers from freely customizable monomers is advantageous for achieving compatibility of spinning strategies with existing hydrogel design strategies. Hence, the most common AM was selected as the base monomer to study the continuous spinning strategy of hydrogel fibers from the monomers. Interestingly, we found that PAM hydrogel fibers prepared by ultraviolet (UV)‐initiated AM polymerization were easily self‐lubricating out of the fluorinated ethylene propylene (FEP) tubular mold and left a lubricating solution, while the PAM hydrogel fiber prepared in the glass tubular mold was difficult to push out (**Figure** **1**a). To investigate the reason for the PAM hydrogel fiber self‐lubricating out of the FEP mold, the components and origin of the lubricating solution were studied. Drying weighing and FTIR tests showed that the lubricating solution was a mixture of mostly water and a little partially polymerized AM (Figures S1 and S2, Supporting Information). Therefore, we hypothesized that the lubricating solution originated from the inhibition of monomer polymerization by the FEP mold.

To exclude the influence factors other than molds, the vertical UV polymerization experiment was employed to investigate the effect of the FEP tubular and glass tubular molds on the monomer polymerization into a hydrogel (Figure 1b). After 2 min of UV polymerization, a solid‐liquid mixed system with a central gel phase and a liquid phase around the mold wall was formed in the hydrophobic FEP mold, while a wall‐fitted hydrogel was formed in the hydrophilic glass mold (Figure 1c–f). Subsequently, the relationship between polymerization time and hydrogel size was studied by using FEP/glass molds with different internal diameters (Figure 1g). The hydrogels in FEP molds of various internal diameters all grew rapidly to a size that was ≈0.55 mm from the inner wall under 1–2 min of UV irradiation, and then the growth rate gradually slowed down during 2–9 min of UV irradiation (Figure 1h). In the glass molds with different internal diameters, the hydrogel size all grew up to close to the inner diameter of the mold at 2 min of UV irradiation. The different results confirmed that the FEP mold could induce mold‐size‐independent regional heterogeneous polymerization of AM solution. Notably, the regional heterogeneous polymerization was also shown in other hydrophobic materials such as silica gel and polytetrafluoroethylene (Figure S3, Supporting Information).

For the regional heterogeneous polymerization induced by hydrophobic materials, Gong and co‐workers attributed it mainly to the oxygen being trapped in the interface between the monomer solution and the rough hydrophobic material (Figure 1i).^[^ ^11^ ^]^ We provided direct support for the view of oxygen inhibition by microscopic observation. As shown in Figure 1j, there were dense and small gaps at the interface between the FEP mold and the monomer solution, while there were no visible gaps at the interface between the glass mold and the monomer solution. These gaps provided space for oxygen to remain, which led to the inhibition of the polymerization of the monomer solution into a hydrogel by oxygen. Furthermore, the relationship between oxygen permeability and oxygen inhibition effect in the FEP mold was studied. The increase in the outer diameter of the FEP mold did not significantly affect the size of the gel phase, implying that oxygen permeation of the FEP tube was not the main reason for the oxygen inhibition effect (Figure S4, Supporting Information). To summarize, we could establish a self‐lubrication model that described the process of the monomer solution polymerized into the hydrogel fiber and self‐lubricating out from the hydrophobic mold (Figure 1k). Briefly, under UV irradiation, the hydrogel fiber was formed in the center of the hydrophobic mold, and the lubricating solution containing partially polymerized monomers was generated near the tubular mold wall due to the regional heterogeneous polymerization induced by hydrophobic mold. The lubricating solution could effectively avoid the contact between the hydrogel fiber and the hydrophobic mold wall, thus allowing the hydrogel fiber to successfully self‐lubricating out of the hydrophobic mold after applying a small stress.

### 2.2 Continuous Spinning of Hydrogel Fibers by Using the SLS Strategy

**Figure 2:**
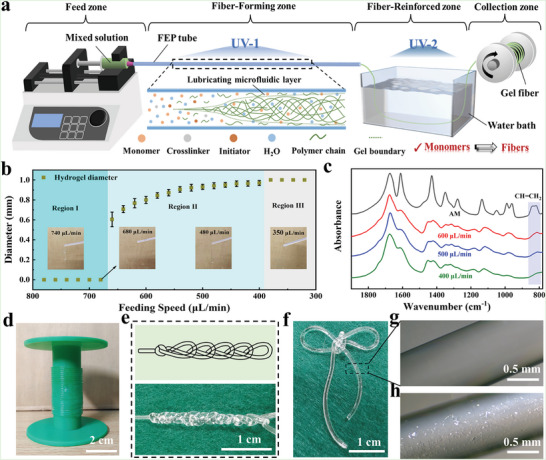
a) A continuous spinning system based on the SLS strategy. Effects of feed speed on b) the phase state and c) polymerization degree of spinning products. d) PAM hydrogel fibers spun by the SLS strategy. e,f) PAM hydrogel fibers‐based knitted fabrics. Optical images of PAM hydrogel fibers prepared by the g) SLS strategy and h) template‐extrusion method.

Based on the self‐lubrication model, a continuous spinning system was constructed for fabricating hydrogel fibers from monomers. The continuous spinning system included the feed zone, fiber‐forming zone, fiber‐reinforced zone, and collection zone (**Figure** **2**a). The syringe pump in the feed zone provided a stable force to transform the static lubricating solution generated in the fiber‐forming zone into a lubricated microfluidic layer for the smooth spinning of hydrogel fibers. And hydrogel fibers increased their polymerization degree in the fiber‐reinforced zone before being collected. A mixed solution of AM, N, N′‐methylene bisacrylamide (MBA), 2‐hydroxy‐2‐methylpropiophenone (I1173), and deionized water was prepared for the continuous spinning of PAM hydrogel fibers and the subsequent study of spinning parameters. The effect of feed speed on the spinning product phase state is presented in Figure 2b. As the feed speed decreased, the spinning product transformed from the liquid phase to the gel phase, and the size of the gel phase gradually increased until the hydrogel fiber was blocked in the FEP tube after the feed speed was less than 400 µL min^−1^. Depending on the phase state of the spinning product and whether it was blocked in the FEP tube, three speed regions could be divided, including Region I, Region II, and Region III. Obviously, ideal hydrogel fibers could be obtained only when the feed speed was within Region II (Movie S1, Supporting Information). Then, the effect of the feed speed on the polymerization degree and mechanical property of obtained hydrogel fibers was roughly estimated by FTIR and tensile tests. As shown in Figure 2c and Figures S5 and S6 (Supporting Information), as the feed speed was reduced from 600 to 400 µL min^−1^, the C = C conversion rate of obtained hydrogel fibers increased from 53.93% to 83.75%, and its mechanical properties became better. Clearly, the size, polymerization degree, and mechanical property of the obtained hydrogel fibers could be adjusted by regulating the feed speed.

Moreover, the changes in the spinnable interval (Region II) of the SLS strategy were studied by drastically varying the polymerization kinetic parameters such as monomer concentration, crosslinker concentration, initiator concentration, and UV distance. As shown in Figure S7 (Supporting Information), the starting and end points of Region II were shifted nearly linearly toward higher feed speeds as the concentrations of AM, MBA, and I1173 increased. In contrast, with the UV distance increased, the starting and ending points of Region II shift almost linearly toward lower feed speeds. Obviously, the spinnability of the SLS strategy was related to the polymerization kinetic parameters of hydrogel fibers, without the need for specific rheological properties of the spinning solution.

Figure 2d–f displayed images of PAM hydrogel fibers fabricated by the SLS strategy, which could be knitted for practical and interesting knitted fabrics (Movies S2 and S3, Supporting Information). Additionally, owing to the protection of the lubricating layer generated by the regional heterogeneous polymerization, the PAM hydrogel fiber prepared by the SLS strategy exhibited fewer surface defects compared to the PAM hydrogel fiber prepared by the template‐extrusion method (Figure 2g,h).

### 2.3 Monomer Universality and Hydrogel Design Strategy Compatibility of the SLS Strategy

**Figure 3:**
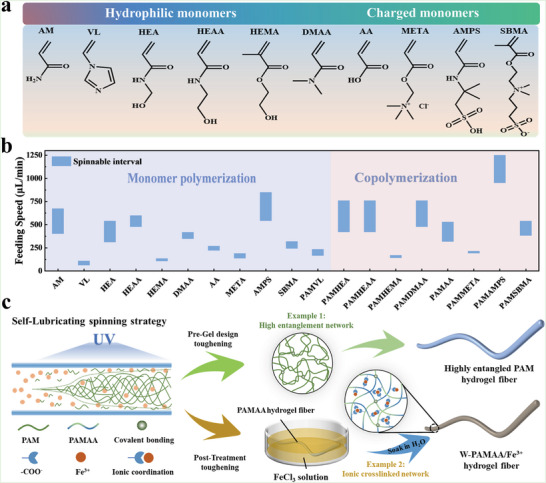
a) Ten vinyl monomers were used to demonstrate the universality of the SLS strategy. b) Spinnable intervals of hydrogel fibers prepared from ten vinyl monomers or AM copolymerization with other monomers. c) The SLS strategy combined with highly entangled and ionic crosslinked hydrogel design strategies to prepare high‐tough hydrogel fibers.

An ideal hydrogel fiber spinning strategy should be universal and compatible with many of the existing hydrogel design strategies. Therefore, ten common vinyl monomers were used to prepare the spinning solution for demonstrating the universality of the SLS strategy (**Figure** **3**a). As shown in Figure 3b, all monomers could be successfully spun into hydrogel fibers, and they exhibited inconsistent spinnable intervals due to differences in their physicochemical properties. Notably, the AM could also be copolymerized with other monomers to introduce functional groups such as imidazole groups, carboxyl groups, sulfonic acid groups, etc. to achieve multifarious designs of hydrogel fibers.

Therefore, the SLS strategy allowed the network structure to be designed freely for endowing the hydrogel fibers with desired performances such as high mechanical properties, conductivity, and anti‐swelling properties. As shown in Figure 3c, the SLS strategy allowed adjusting the monomer and crosslinker concentrations of the spinning solution to directly spin high‐tough PAM hydrogel fibers with a highly entangled network.^[^ ^12^ ^]^ In addition, existing post‐toughening strategies for hydrogels could be compatible with the SLS strategy. The poly(acrylamide‐*co*‐acrylic acid) (PAMAA) hydrogel fiber with carboxyl groups was purposely spun by using the SLS strategy. Then, the PAMAA hydrogel fiber was soaked into the FeCl_3_ solution and toughened by utilizing the coordination of carboxylic acid with Fe^3+^ to obtain the poly(acrylamide‐*co*‐acrylic acid)/Fe^3+^ (PAMAA/Fe^3+^) hydrogel fiber. Further, the PAMAA/Fe^3+^ hydrogel fibers were soaked in H_2_O to remove the excess counteracting ions to achieve the high‐tough W‐PAMAA/Fe^3+^ hydrogel fiber with an ionic crosslinking network.^[^ ^13^ ^]^

### 2.4 Mechanical Properties and Toughening Mechanism of High‐Tough Hydrogel Fibers Fabricated by the SLS Strategy

**Figure 4:**
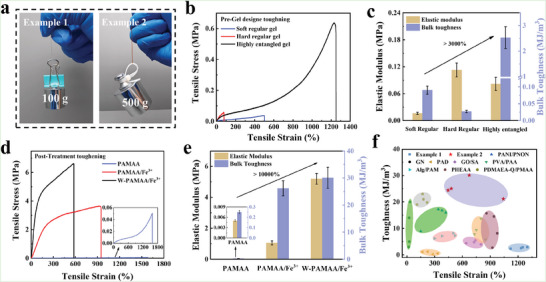
a) The PAM and W‐PAMAA/Fe^3+^ hydrogel fibers were able to lift 100 g and 500 g weights, respectively. Toughening effect of b,c) high entanglement strategy and d,e) ionic crosslinked strategy. f) Ashby plots of toughness and tensile strain values of highly entangled PAM, W‐PAMAA/Fe^3+^, and reported other hydrogel fibers.

Mechanical properties of highly entangled PAM and ionic crosslinked W‐PAMAA/Fe^3+^ hydrogel fibers were shown in **Figure** **4**a, they could easily lift 100 g and 500 g weights, respectively. After component optimization, the toughness of the highly entangled PAM hydrogel fibers could reach 2.53 ± 0.43 MJ m^−3^, which was improved by ≈30 times compared to the soft regular and hard regular PAM hydrogel fibers (Figure S8, Supporting Information; Figure 4b,c). The optimized ionic crosslinked W‐PAMAA/Fe^3+^ hydrogel fiber with a toughness of 30.08 ± 3.88 MJ m^−3^, which was approximately 100 times higher than the PAMAA hydrogel fiber (Figure S9, Supporting Information; Figure 4d,e). The remarkable toughening effect implied that the SLS strategy could be effectively compatible with existing hydrogel design strategies. It was notable that the mechanical properties of the obtained W‐PAMAA/Fe^3+^ hydrogel fiber were stronger than most of the reported hydrogel fibers (Figure 4f).^[^ ^5^, ^10^, ^14^ ^]^ Unfortunately, the detailed toughening mechanisms for Fe^3+^ crosslinked PAMAA hydrogel systems were lacking, and researchers seem to be more concerned with the changes in mechanical properties of the hydrogel.^[^ ^13^, ^15^ ^]^

**Figure 5:**
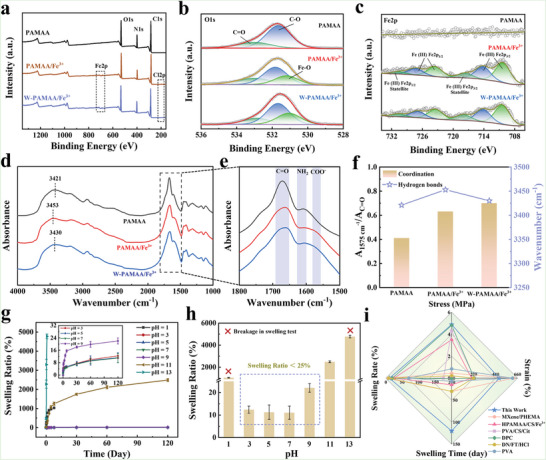
a) Total XPS spectrum, b) O1s spectrum, c) Fe2p spectrum, and d,e) FTIR of PAMAA, PAMAA/Fe^3+^, and W‐PAMAA/Fe^3+^ hydrogel fibers. f) Changes of hydrogen bond and ionic coordination during the preparation of PAMAA hydrogel fiber into W‐PAMAA/Fe^3+^ hydrogel fiber. g,h) Swelling behaviors of W‐PAMAA/Fe^3+^ hydrogel fibers in an environment with a pH of 1–13. i) The comparison of the anti‐swelling properties of W‐PAMAA/Fe^3+^ hydrogel fibers with other anti‐swelling hydrogels.

The toughening mechanism of W‐PAMAA/Fe^3+^ hydrogel fibers was illustrated by comparing the changes in elemental composition, hydrogen bonding interaction, and ionic coordination during the process of preparing the PAMAA hydrogel fiber into the W‐PAMAA/Fe^3+^ hydrogel fiber. As shown in **Figure** **5**a, when the PAMAA hydrogel fiber was soaked in FeCl_3_ solution, the Fe2p and Cl2p characteristic peaks emerged in the PAMAA/Fe^3+^ hydrogel fiber, while the Cl2p characteristic peak in the W‐PAMAA/Fe^3+^ hydrogel fiber disappeared after soaking the PAMAA/Fe^3+^ hydrogel fiber in H_2_O (Figure S10, Supporting Information). It indicated that the element Fe was confined in the W‐PAMAA/Fe^3+^ hydrogel, while the element Cl was removed after soaking in H_2_O. The appearance of Fe‐O fitted peaks in the O1s spectrum and the Fe^3+^ characteristic peaks in the Fe2p spectrum confirmed the formation of carboxylic acid‐Fe^3+^ coordination in PAMAA/Fe^3+^ and W‐PAMAA/Fe^3+^ hydrogel fibers.^[^ ^16^ ^]^ Changes in hydrogen bonding and ionic coordination during the preparation of the PAMAA hydrogel fiber into the W‐PAMAA/Fe^3+^ hydrogel fiber were displayed in Figure 5d. The active hydrogen characteristic peaks of PAMAA, PAMAA/Fe^3+^, and W‐PAMAA/Fe^3+^ hydrogel fibers were located at 3421 cm^−1^, 3451 cm^−1^, and 3430 cm^−1^, respectively, implying that the hydrogen bonding interactions in the hydrogel fiber weakened after soaking in FeCl_3_ solution and were restored after further immersion in H_2_O.^[^ ^17^ ^]^ The 1575 cm^−1^ was assigned to the COO^−^ characteristic peak that could be used to clarify the coordination of COO^−^ with Fe^3+^ (Figure 5e).^[^ ^3^ ^]^ The ratio of absorbance at 1575 cm^−1^ to C = O characteristic peak (A_1575_/A_C = O_) of PAMAA/Fe^3+^ and W‐PAMAA/Fe^3+^ hydrogels were 0.63 and 0.70, respectively (Figure 5f). The increase in A_1575_/A_C = O_ was suggestive of enhanced ionic coordination of the PAMAA/Fe^3+^ hydrogel fiber after soaking in H_2_O due to the removal of counteracting Cl^−^. Benefiting from the strong hydrogen bonding interactions and ionic coordination of the W‐PAMAA/Fe^3+^ hydrogel fiber, it exhibited less than 25% swelling ratio after 120 days of swelling in an environment with a pH of 3–9 (Figure 5g,h). And swollen W‐PAMAA/Fe^3+^ hydrogel fibers still had excellent mechanical properties (tensile stress > 4 MPa, tensile strain > 400%) (Figure S11, Supporting Information; Figure 5i).^[^ ^18^ ^]^ Moreover, The environmental resistance of the obtained hydrogel fibers was investigated. As shown in Figure S12 (Supporting Information), the crystallization peak of the PAMAA hydrogel fiber was −17.9 °C, while that of the W‐PAMAA/Fe^3+^ hydrogel fiber was reduced to −23.7 °C due to the introduction of Fe^3+^.

### 2.5 Multidimensional Sensors Constructed Bottom‐Up with W‐PAMAA/Fe^3+^ Hydrogel Fibers

**Figure 6:**
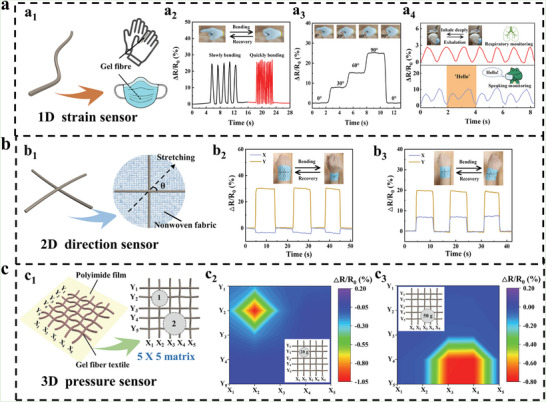
a) The application of the 1D strain sensor based on the W‐PAMAA/Fe^3+^ hydrogel fiber. (a_1_) The 1D strain sensor was integrated into a glove or mask. The 1D strain sensor monitored (a_2_) fast or slow finger bending, (a_3_) finger bending angle, and (a_4_) human breathing and speaking. b) The application of the 2D direction sensor based on the W‐PAMAA/Fe^3+^ hydrogel fiber. (b_1_) The structure of the 2D direction sensor. (b_2‐3_) The 2D direction sensor for monitoring the direction of wrist movement. c) The application of the 3D pressure sensor based on the W‐PAMAA/Fe^3+^ hydrogel fiber. (c_1_) The structure of the 3D pressure sensor. (c_2‐3_) The 3D pressure sensor to achieve the detection of pressure level and pressure position.

The 1D nature of hydrogel fibers allowed them to be weaved and assembled to construct multidimensional sensors from the bottom up. Accordingly, the 1D strain sensor, 2D direction sensor, and 3D pressure sensor were fabricated based on W‐PAMAA/Fe^3+^ hydrogel fibers, respectively. Firstly, due to the conductivity of 0.84 mS cm^−1^ and stretchability of the W‐PAMAA/Fe^3+^ hydrogel fiber, it could be used as a 1D strain sensor by directly connecting electrodes (Figure S13a, Supporting Information). The W‐PAMAA/Fe^3+^ hydrogel fiber‐based strain sensor with a maximum gauge factor (GF) of 4.63 and a detection range of 600%, which was better than the reported hydrogel‐based flexible sensors (Figure S13b,c, Supporting Information).^[^ ^19^ ^]^ The fast response and cyclic stability of the hydrogel fiber‐based strain sensor were provided in Figure S13d–f (Supporting Information). The response and recovery delays of our hydrogel fiber‐based strain sensor were ∼320 ms and ∼300 ms, respectively (Figure S13d, Supporting Information). Such rapid response facilitated real‐time monitoring of applied strain. As shown in Figure S13e,f (Supporting Information), the hydrogel fiber‐based strain sensor exhibited a stable relative resistance change (ΔR/R_0_) at 10–50% strain, and still maintained a good response after 500 s cycle at 50% strain. The results indicated that as the strain increased, the ΔR/R_0_ increased accordingly with good repeatability. The baseline fluctuations of long‐term cycling could be attributed to the accumulation of non‐conductive polymer chains and the decrease in electrical resistance due to prolonged cyclic stretching and evaporation of water from the hydrogel fiber‐based sensor. In addition, the sensor could be integrated into a glove or mask to monitor fast or slow finger bending, finger bending angle, human breathing, and speaking (**Figure** **6**a). Apparently, the W‐PAMAA/Fe^3+^ hydrogel fiber‐based 1D strain sensor not only exhibited good sensing performance, but also could be integrated with other fabrics by utilizing the 1D nature of hydrogel fibers for human behavior monitoring.

The isotropy of conventional hydrogel‐based sensors prevented them from detecting the stress direction, while the 1D nature of hydrogel fibers could amplify the difference in deformation caused by stress in different directions, showing great potential in directional sensing. Therefore, two W‐PAMAA/Fe^3+^ hydrogel fibers were embedded orthogonally in the nonwoven to assemble the 2D direction sensor (Figure S14a, Supporting Information). The 2D direction sensor was stretched at different angles, and the ΔR/R_0_ of each of the two fibers was captured for evaluating the direction sensing performance of the 2D direction sensor (Figure S14b–d, Supporting Information). It could be seen that as the angle increases from 0° to 45°, the GF of the X‐axis decreased from 1.54 to 0.53 and the GF of the Y‐axis increased from −0.095 to 0.53, exhibiting obvious direction‐induced GF changes (ΔGF). Figure S14e (Supporting Information) presented a model that showed the ΔR/R_0_ during the interaction of tensile angle and strain, which was used to obtain the tensile strain and angle for a given ΔR/R_0_. The directional selectivity factor referred to the magnitude of the ΔGF when the stretching angle changed, and it was used to evaluate the directional selectivity of the 2D direction sensor. The directional selectivity factor of the 2D direction sensor was −0.037, which could effectively distinguish the direction of the applied load (Figure S14f, Supporting Information). As shown in Figure 6b, the 2D direction sensor was used for monitoring the direction of wrist movement. When the wrist was bent back and forth, the ΔR/R_0_ of the X and Y axes was −3.48% and 29.97%, while when the wrist was bent left and right, the ΔR/R_0_ of the X and Y axes were 6.95% and 19.63%. The anisotropy of hydrogel fibers effectively amplified the differences in electrical signals induced by external stimuli, and thus the strain direction was easily discerned by the fiber‐based 2D direction sensor.

In addition, a basic pressure‐sensing unit could be fabricated by connecting the positive and negative electrodes to a W‐PAMAA/Fe^3+^ hydrogel fiber and another same fiber orthogonal to it, respectively. When the pressure‐sensing unit was pressurized, the contact surface of the two fibers increased, resulting in a decrease in the electrical resistance, thus enabling the conversion of pressure‐to‐electric signals (Figure S15a, Supporting Information). The sensitivity, response delay, and recovery delay of the pressure‐sensing unit were 0.00024 KPa^−1^, ≈200 ms, and ≈290 ms, respectively (Figure S15b,c, Supporting Information). And the pressure‐sensing unit displayed a stable ΔR/R_0_ at pressures of 10–90 KPa (Figure S15d,e, Supporting Information). Furthermore, a plurality of pressure‐sensing units could be weaved and assembled to construct a 3D pressure sensor with the 5 × 5 pressure‐sensing unit array (Figure 6c** _1_**). The 3D pressure sensor could not only respond to the pressure level, but also detect the pressure position. As shown in Figure 6c** _2‐3_**, when a weight of 20 g was placed in the upper left corner of the 3D pressure sensor, an ΔR/R_0_ of ≈−1.02% occurred around coordinate (X_2_, Y_2_), while there was no significant resistance change in the other coordinates. When a weight of 50 g was placed in the lower right corner of the sensor, the ΔR/R_0_ of ≈−0.77% was exhibited around coordinates (X_3_, Y_4_), (X_3_, Y_5_), (X_4_, Y_4_), and (X_4_, Y_5_) due to the pressure involving multiple pressure‐sensing units. It was easy to see that the 1D nature of hydrogel fibers allowed them to construct the desired 3D structures bottom‐up by weaving and assembling to achieve specific sensing functions.

**Figure 7:**
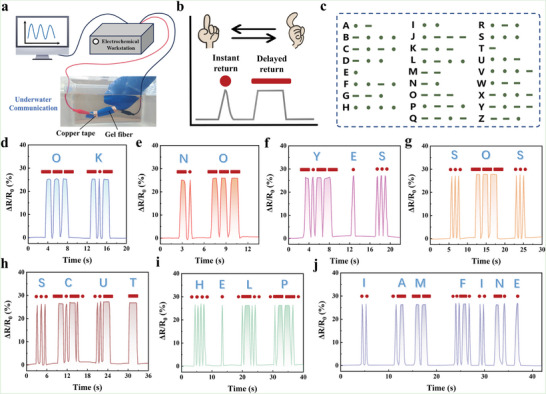
a) The W‐PAMAA/Fe^3+^ hydrogel fiber‐based sensor for underwater communication. b) The triangular and quasi‐rectangular electrical signal were obtained by finger bending. c) Morse code table. d–j) The W‐PAMAA/Fe^3+^ hydrogel fiber‐based strain sensor output words and sentences such as “OK”, “NO”, “YES”, “SOS”, “SCUT”, “HELP”, and “I AM FINE”.

On the other hand, the W‐PAMAA/Fe^3+^ hydrogel fiber could be used as an underwater communication sensor due to its excellent anti‐swelling properties (**Figure** **7**a). The triangular electrical signal formed by the immediate return of the bent finger was defined as a point, and the quasi‐rectangular electrical signal formed by the delayed return of the bent finger was defined as a bar (Figure 7b). According to the Morse code table, various words could be output by the combination of points and bars (Figure 7c). For example, three quasi‐rectangular electrical signals could be translated as “O”, and two quasi‐rectangular electrical signals sandwiched between a triangular electrical signal translated as “K”, thereby outputting the “OK” word (Figure 7d). By the same way, words and sentences such as “NO”, “YES”, “SOS”, “SCUT”, “HELP”, and “I AM FINE” could be output for underwater communication (Figure 7e–j). Clearly, benefiting from the compatibility of the SLS strategy with existing hydrogel network design strategies, hydrogel fibers could be endowed with multiple functions for application in specific environments.

## 3 Conclusion

In this study, we found that hydrophobic mold induced regional heterogeneous polymerization enabled the vinyl monomer solution to form a structure with a gel phase in the mold center and a lubricating liquid phase around the mold wall during UV polymerization into the hydrogel fiber, thus allowing the hydrogel fiber to self‐lubricate out of hydrophobic tubular molds. Therefore, a continuous spinning system based on the SLS strategy was constructed for achieving continuous spinning from monomers to hydrogel fibers. The size and polymerization degree of the obtained hydrogel fibers were manageable by adjusting the feed speed. In addition, ten common vinyl monomers could successfully spin hydrogel fibers by the SLS strategy, confirming the universality of the SLS strategy. Benefiting from the universality of the SLS strategy, the SLS strategy could be compatible with existing hydrogel design strategies to fabricate hydrogel fibers with excellent mechanical properties, such as highly entangled PAM and ionic crosslinked W‐PAMAA/Fe^3+^ hydrogel fibers. The tensile stress/strain of resulted PAM and W‐PAMAA/Fe^3+^ hydrogel fibers were 0.63 ± 0.42 MPa/1229.02 ± 80.62% and 6.61 ± 0.38 MPa/577.97 ± 40.61%, respectively. Notably, the W‐PAMAA/Fe^3+^ hydrogel fiber exhibited long‐term mechanical stability after 120 days of swelling in an environment with a pH of 3–9 due to the synergetic effect of strong hydrogen bond interaction and ionic coordination. Furthermore, W‐PAMAA/Fe^3+^ hydrogel fibers were weaved and assembled bottom‐up to fabricate the 1D strain sensor, 2D direction sensor, and 3D pressure sensor for human behavior monitoring, pressure level/pressure position detection, and underwater communication. The successful fabrication and application of these hydrogel fiber‐based sensors demonstrated the tremendous advantages of hydrogel fibers with 1D nature for flexible electronics applications, including the ability to integrate into other fabrics, amplify differences in electrical signals caused by strain with different directions, and self‐weave into complex multidimensional structures.

## 4 Experimental Section

### Materials

Acrylic amide (AM), acrylic acid (AA), N‐hydroxymethyl acrylamide (HEA), 2‐hydroxy‐2‐methylpropiophenone (I1173), and FeCl_3_ were purchased from Shanghai Macklin Biochemical Co., Ltd. Vinyl imidazole (VL), 2‐acrylamido‐2‐methylpropane sulfonic acid (AMPS), hydroxyethyl methacrylate (HEMA), N, N′‐methylene bis (acrylamide) (MBA) were purchased from Shanghai Aladdin Bio‐Chem Technology Co., Ltd. N, N‐Dimethyl‐2‐propenamide (DMAA), N‐hydroxyethyl acrylamide (HEAA), and methacryloxyethyltrimethyl ammonium chloride (META) were purchased from Shanghai Bide Medical Technology Co., Ltd. The sulfobetaine methacrylate (SBMA) was purchased from Shanghai Haohong Biomedical Technology Co., Ltd. The deionized water was obtained using a water purification system.

### Fabrication of PAM‐Based Hydrogel Fibers by the SLS strategy

For the highly entangled PAM hydrogel fiber, 6 mol L^−1^ AM, I1173 (1 mol% of monomer), and MBA (0.01 mol% of monomer) were H_2_O‐sized to 15 mL as the spinning solution. The spinning solution was pushed by a syringe pump at a feed speed of 1150 µL min^−1^ into the FEP tube with an inner diameter of 1 mm. The spinning solution in the FEP tube was irradiated by a 365 nm UV light with a power of 10 W to initiate free radical polymerization. The PAM hydrogel fiber was formed in the middle of the FEP tube, and a partially polymerized lubricating solution was generated near the tube wall to prevent contact between the hydrogel fiber and the FEP tube wall for continuous spinning of the PAM hydrogel fiber. The spun‐out PAM hydrogel fiber was reinforced by a 50 W 365 nm UV light, and then it was collected by a collection roller that had a collection speed similar to the feed speed.

For the soft regular PAM hydrogel fiber, 3 mol L^−1^ AM, I1173 (1 mol% of monomer), and MBA (0.1 mol% of monomer) were H_2_O‐sized to 15 mL as the spinning solution. The feed speed was 450 µL min^−1^, and other parameters were consistent with the fabrication of the highly entangled hydrogel.

For the hard regular PAM hydrogel fiber, 3 mol L^−1^ AM, I1173 (1 mol% of monomer), MBA (1 mol% of monomer) were H_2_O‐sized to 15 mL as the spinning solution. The feed speed was 550 µL min^−1^, and other parameters were consistent with the fabrication of the highly entangled hydrogel.

### Fabrication of W‐PAMAA/Fe^3+^ Hydrogel Fibers by the SLS Strategy

For the PAMAA hydrogel fiber, 2.55 mol L^−1^ AM, 0.45 mol L^−1^ AA, I1173 (1 mol% of total monomer), and MBA (0.01 mol% of total monomer) were H_2_O‐sized to 15 mL as the spinning solution. The feed speed was 320 µL min^−1^. The spinning solution in the FEP tube was irradiated by a 365 nm UV light with a power of 10 W to initiate free radical polymerization. The spun‐out PAMAA hydrogel fiber was reinforced by a 50 W 365 nm UV light, and then it was collected by a collection roller that had a collection speed similar to the feed speed. The obtained PAMAA hydrogel fiber was soaked in 0.05 mol L^−1^ FeCl_3_ solution for 6 h to prepare the PAMAA/Fe^3+^ hydrogel fiber. Next, the PAMAA/Fe^3+^ hydrogel fiber was soaked in H_2_O for 24 h to achieve W‐PAMAA/Fe^3+^ hydrogel fiber.

### Informed Consent

Experimental data regarding human subjects were provided after informed consent was obtained from volunteers. It was confirmed that the use of flexible electronics in the study did not require the approval of an institutional review board.

## Conflict of Interest

The authors declare no conflict of interest.

## Supporting information

Supporting Information

Supplemental Movie 1

Supplemental Movie 2

Supplemental Movie 3

## Data Availability

Research data are not shared.

## References

[1] a) Y. Ohm , C. Pan , M. J. Ford , X. Huang , J. Liao , C. Majidi , Nat. Electron. 2021, 4, 185;

[2] a) X. Xiong , Y. Chen , Z. Wang , H. Liu , M. Le , C. Lin , G. Wu , L. Wang , X. Shi , Y. G. Jia , Y. Zhao , Nat. Commun. 2023, 14, 1331; 36898994 10.1038/s41467-023-36920-3 PMC10006079

[3] M. Ju , B. Wu , S. Sun , P. Wu , Adv. Funct. Mater. 2020, 30, 1910387.

[4] J. Cheng , Y. Jun , J. Qin , S. H. Lee , Biomaterials 2017, 114, 121. 27880892 10.1016/j.biomaterials.2016.10.040

[5] a) C. K. Chu , A. J. Joseph , M. D. Limjoco , J. Yang , S. Bose , L. S. Thapa , R. Langer , D. G. Anderson , J. Am. Chem. Soc. 2020, 142, 19715; 33141568 10.1021/jacs.0c09691 PMC9455704

[6] a) A. Sydney Gladman , E. A. Matsumoto , R. G. Nuzzo , L. Mahadevan , J. A. Lewis , Nat. Mater. 2016, 15, 413; 26808461 10.1038/nmat4544

[7] a) J. Song , S. Chen , L. Sun , Y. Guo , L. Zhang , S. Wang , H. Xuan , Q. Guan , Z. You , Adv. Mater. 2020, 32, 1906994; 10.1002/adma.201906994 31957099

[8] X. Y. Du , Q. Li , G. Wu , S. Chen , Adv. Mater. 2019, 31, 1903733. 10.1002/adma.201903733 31573714

[9] X. Duan , J. Yu , Y. Zhu , Z. Zheng , Q. Liao , Y. Xiao , Y. Li , Z. He , Y. Zhao , H. Wang , L. Qu , ACS Nano 2020, 14, 14929. 33073577 10.1021/acsnano.0c04382

[10] a) L. Zhao , T. Xu , B. Wang , Z. Mao , X. Sui , X. Feng , Chem. Eng. J. 2023, 455, 140796;

[11] a) M. Peng , T. Kurokawa , J. P. Gong , Y. Osada , Q. Zheng , J. Phys. Chem. B 2002, 106, 3073;

[12] J. Kim , G. Zhang , M. Shi , Z. Suo , Science 2021, 374, 212. 34618571 10.1126/science.abg6320

[13] P. Lin , S. Ma , X. Wang , F. Zhou , Adv. Mater. 2015, 27, 2054. 25677412 10.1002/adma.201405022

[14] a) T. Chen , P. Wei , G. Chen , H. Liu , I. T. Mugaanire , K. Hou , M. Zhu , J. Mater. Chem. A 2021, 9, 12265; 10.1021/acsami.9b20254 31869196

[15] Y. Hu , Z. Du , X. Deng , T. Wang , Z. Yang , W. Zhou , C. Wang , Macromolecules 2016, 49, 5660.

[16] a) Z. Xu , J. Li , G. Gao , Z. Wang , Y. Cong , J. Chen , J. Yin , L. Nie , J. Fu , J. Polym. Sci., Part B: Polym. Phys. 2018, 56, 865;

[17] a) H. Sun , Y. Zhao , S. Jiao , C. Wang , Y. Jia , K. Dai , G. Zheng , C. Liu , P. Wan , C. Shen , Adv. Funct. Mater. 2021, 31, 2101696;

[18] a) S. He , X. Sun , Z. Qin , X. Dong , H. Zhang , M. Shi , F. Yao , H. Zhang , J. Li , Adv. Mater. Technol. 2022, 7, 2101343;

[19] a) L. Xu , Z. Huang , Z. Deng , Z. Du , T. L. Sun , Z. H. Guo , K. Yue , Adv. Mater. 2021, 33, 2105306; 10.1002/adma.202105306 34647370

