# Supplementary material for: Continuous Spinning of High‐Tough Hydrogel Fibers for Flexible Electronics by Using Regional Heterogeneous Polymerization
Source: Adv Sci (Weinh). 2023 Oct 27;10(36):2305226. doi: 10.1002/advs.202305226 (PMC10754135; doi:10.1002/advs.202305226)
Supplement: Supplementary file 1 — Supporting Information [file ADVS-10-2305226-s002.pdf]

ADVANCED SCIENCE
Open Access

Supporting Information

for *Adv. Sci.*, DOI 10.1002/advs.202305226

Continuous Spinning of High-Tough Hydrogel Fibers for Flexible Electronics by Using Regional Heterogeneous Polymerization

Shaoji Wu, Caihong Gong, Zichao Wang, Sijia Xu, Wen Feng, Zhiming Qiu and Yurong Yan**

Continuous Spinning of High-Tough Hydrogel Fibers for Flexible Electronics by Using Regional Heterogeneous Polymerization

Shaoji Wu[a], Caihong Gong[a], Zichao Wang[a], Sijia Xu[a], Wen Feng[b], Zhiming Qiu[a,*], Yurong Yan[a, c*]

[a] School of Materials Science and Engineering, South China University of Technology, Guangzhou, 510641, P.R. China.

[b] Guangzhou Fiber Product Testing Institute, Guangzhou, 511447, P.R. China.

[c] Key Lab of Guangdong High Property & Functional Polymer Materials, Guangzhou, 510640, P.R. China

* Corresponding authors E-mail: mszqiu@scut.edu.cn, yryan@scut.edu.cn

Table of contents

Experiment and Method --S-2

Fig. S1 --S-4

Fig. S2 --S-4

Fig. S3 --S-4

Fig. S4 --S-5

Fig. S5 --S-5

Fig. S6 --S-6

Fig. S7 --S-6

Fig. S8 --S-7

Fig. S9 --S-7

Fig. S10 ---S-8

Fig. S11 ---S-8

Fig. S12 ---S-8

Fig. S13 ---S-9

Fig. S14 ---S-9

Fig. S15 --S-10

Experiment and Method

The universality of the SLS strategy: The spinning solution of PAM, PAA, HEA, HEAA, HEMA, and DMAA hydrogel fibers consisted of 3 mol/L monomers, I1173 (1 mol% of monomer), MBA (0.01 mol% of monomer), and 15 mL H_2O. The spinning solution of APMS and SBMA hydrogel fibers consisted of 1 mol/L monomer, I1173 (1 mol% of monomer), MBA (2 mol% of monomer), and 15 mL H_2O. The spinning solution of META hydrogel fiber consisted of 3 mol/L META, I1173 (1 mol% of monomer), MBA (2 mol% of monomer), and 15 mL H_2O. The spinning solution of hydrogel fibers copolymerized with AM and other monomers consisted of 3 mol/L total monomers (85% AM and 15% other monomers), I1173 (1 mol% of total monomer), MBA (1 mol% of total monomer), and 15 mL H_2O. The feed speeds of all hydrogel fibers mentioned above were presented in Fig. 3f, and other parameters were consistent with the fabrication of the highly entangled hydrogel.

FTIR tests: Fourier transform infrared (FTIR) tests of the samples were acquired using a Nicolet IS50-Nicolet Continuum produced by Thermo Fisher Scientific, Ltd., where transmission mode was employed for sample detection.

XPS tests: X-ray photoelectron spectroscopy (XPS) analysis was performed on a Kratos Axis Ultra-DLD X-ray photoelectron spectrometer.

Mechanical properties tests: The mechanical properties of hydrogel fibers were tested by using a universal tester (AGS-X). The stress (σ) was calculated as $\sigma = F/A_0$, where F was the load force, and A_0 was the original cross-sectional area. The strain (ε) was estimated as $\varepsilon = \Delta l/l_0$, where Δl was the length of the stretch and l_0 was the original length. The elastic modulus (E) was obtained by calculating the slope of the stress-strain curve in the region between 0-5% strain. The bulk toughness (W) was obtained as $W = \int_{\varepsilon=0}^{\varepsilon=\varepsilon_b} \sigma_{\mathrm{load}}\, d\varepsilon$, where ε_b and σ_{load} were the corresponding breaking strains and

stresses in the loading process, respectively. Energy dissipation (U_{hys}) was obtained as $U_{hys} = \int_{\varepsilon=0}^{\varepsilon=\varepsilon_x}(\sigma_{load} - \sigma_{unload})\, d\varepsilon$, where ε_x was the preset strain, and σ_{load} and σ_{unload} were the corresponding stresses in the loading and unloading processes, respectively.

Conductivity test: The electrical resistance of hydrogel fiber was measured by using a SourceMeter (Keithley DMM7510). The conductivity (σ) was calculated as $\sigma = \Delta l/(R \times S)$, where Δl, R, and S were the length, electrical resistance, and cross-sectional area of hydrogel fiber, respectively.

Sensing tests of hydrogel fiber-based sensors*:* The sensing tests of the hydrogel fiber-based sensor were carried out by the combination of a universal tensile testing machine (AGS-X) and a CHI660D electrochemical workstation at a constant voltage of 1 V. The gauge factor (GF) was estimated as GF = $\Delta R/R_0/\varepsilon$, where ΔR was the resistance changes with strain, R_0 was the resistance of the hydrogel fiber at the original length, and ε was the applied strain. The sensitivity (S) of the pressure-sensing unit was estimated as S = $\Delta R/R_0/\sigma$, where $\Delta R/R_0$ was the relative resistance changes, σ was the applied stress.

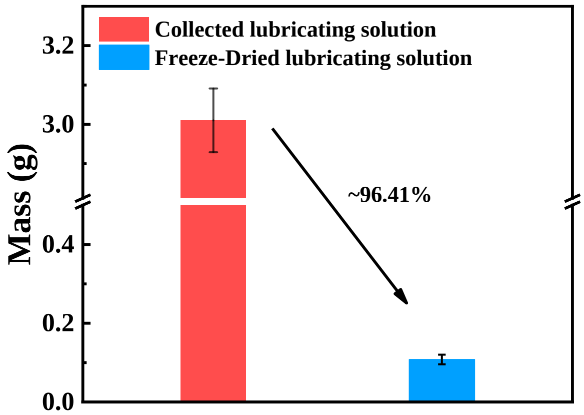

Fig. S1 The drying weighing method to measure the water content of the lubricating solution.

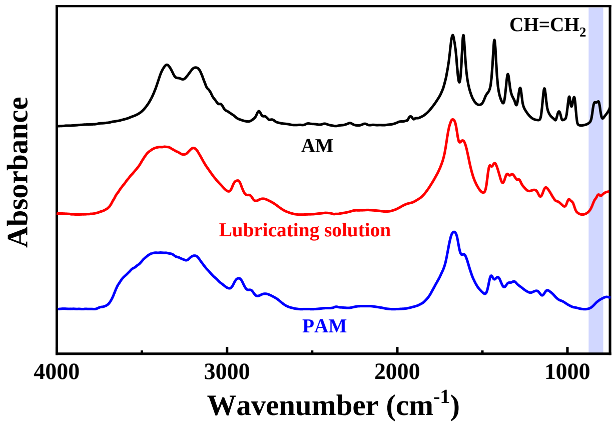

Fig. S2 FTIR tests of AM, lubricating solution, and PAM.

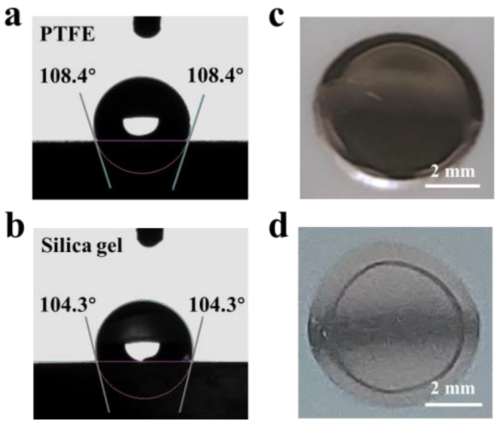

Fig. S3 The hydrophilicity of the (a) polytetrafluoroethylene (PTFE) and (b) silica gel mold. The regional heterogeneity of AM solutions polymerized in the (c) PTFE and (d) silica gel mold.

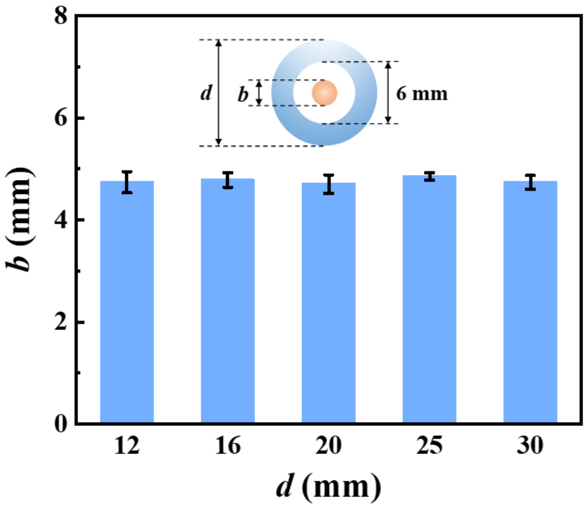

Fig. S4 the relationship between oxygen permeability and oxygen inhibition effect in the FEP bulk.

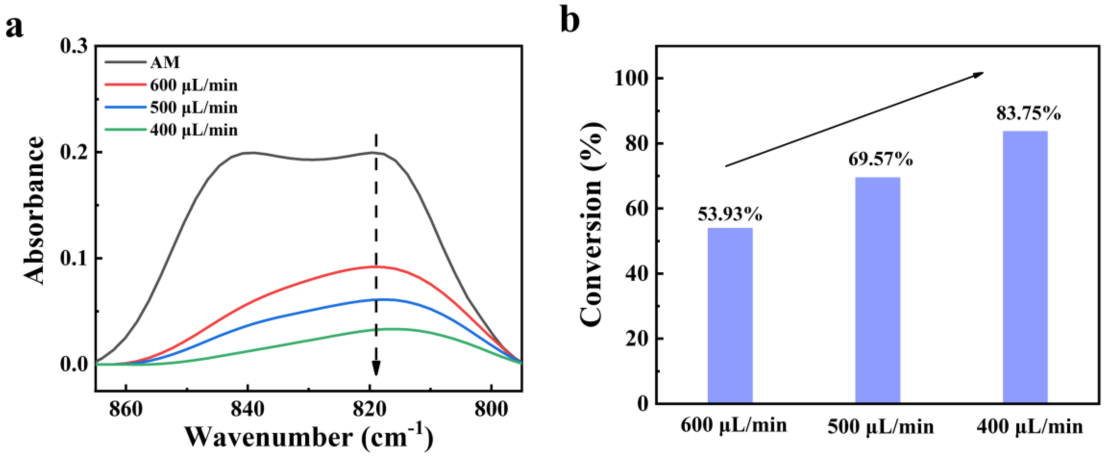

Fig. S5 (a) FTIR of PAM hydrogel fibers fabricated at different feed speeds (normalized with C=O peak at 1675 cm^{-1} was used as the internal standard peak). (b) Monomer conversion rates (*C*) of PAM hydrogel fibers fabricated at different feed speeds. The *C* was calculated based on the following formula.

$$C = (1 - \frac{A_{819}^{fiber}}{A_{819}^{monomer}}) \times 100\%$$

Where A_{819}^{fiber} and $A_{819}^{monomer}$ represented the absorption at 819 cm^{-1} of PAM hydrogel fiber and monomer, respectively.

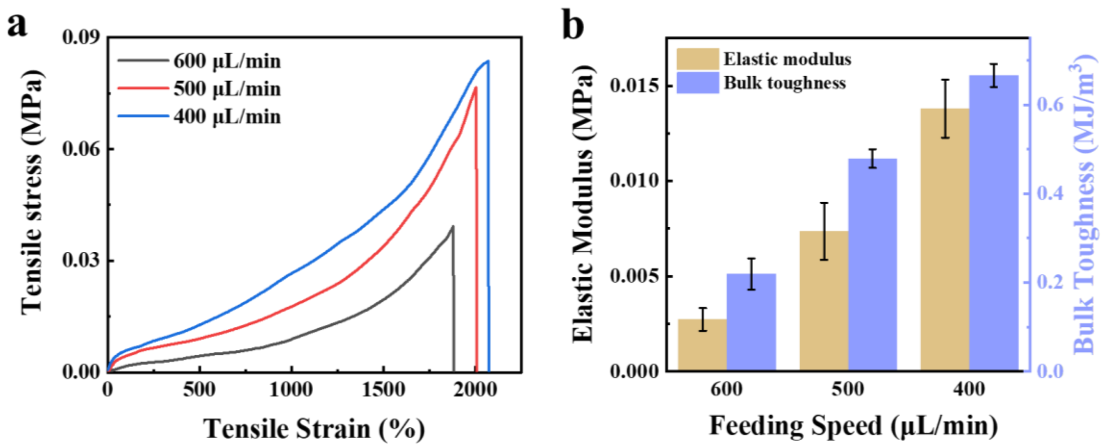

Fig. S6 The effect of the feed speed of the spinning solution on the mechanical properties of PAM hydrogel fibers.

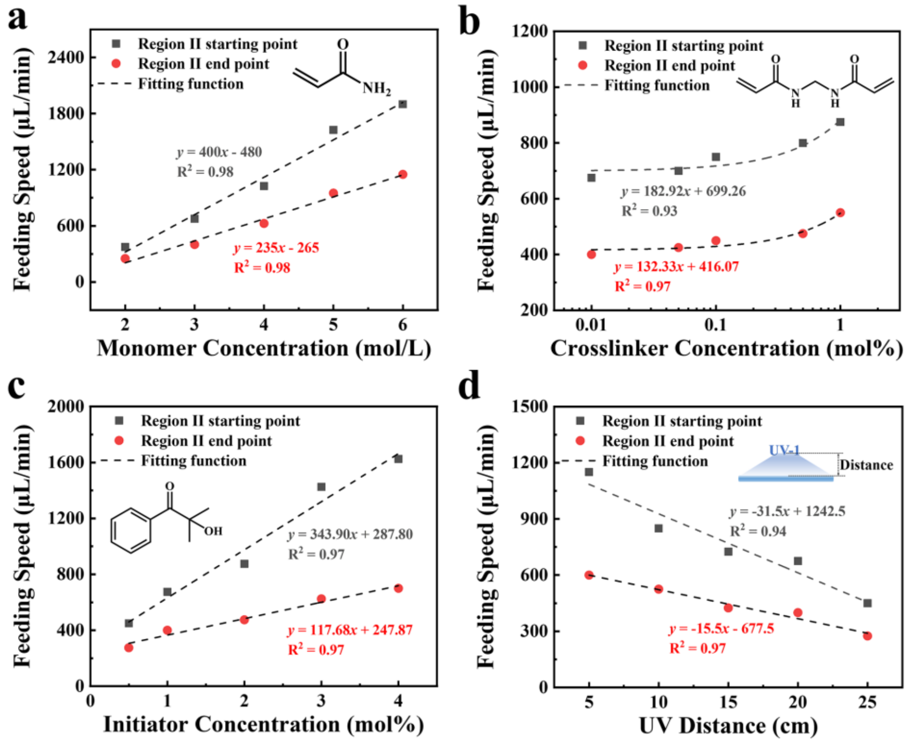

Fig. S7 Effect of varying the (a) monomer concentration, (b) crosslinker concentration, (c) initiator concentration, and (d) UV distance on the starting and ending points of Region II.

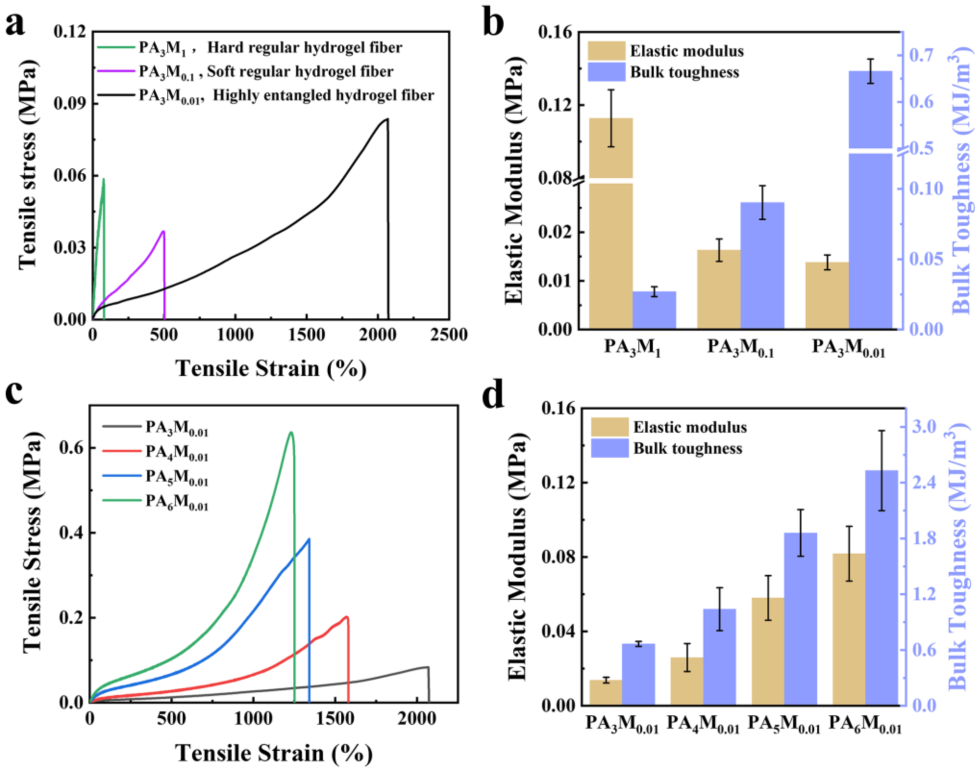

Fig. S8 Effects of the (a) MBA and (b) AM concentration on the mechanical properties of PAM hydrogels. For PA_xM_y, A represented AM, x was the molar concentration of AM, M represented MBA, and y was the molar percentage of MBA relative to AM. The values of y for hard regular PAM hydrogel, soft regular PAM hydrogel and highly entangled PAM hydrogel fibers were 1, 0.1, and 0.01, respectively.

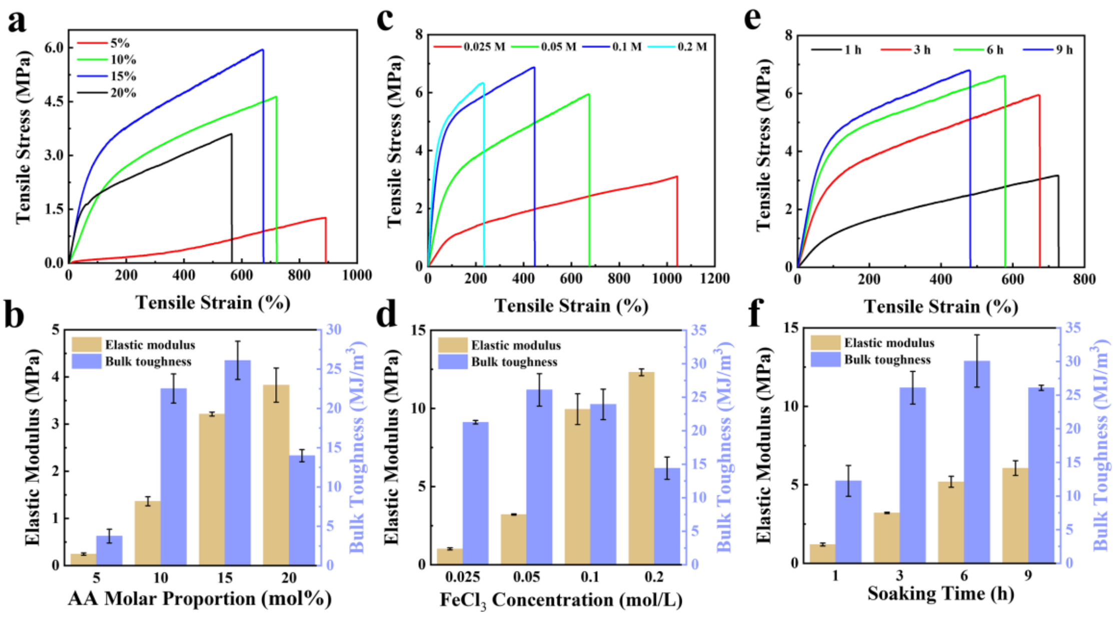

Fig. S9 Effects of the (a) AA concentration, (b) $FeCl_3$ concentration, and (c) soaking time of $FeCl_3$ on the mechanical properties of PAM hydrogels.

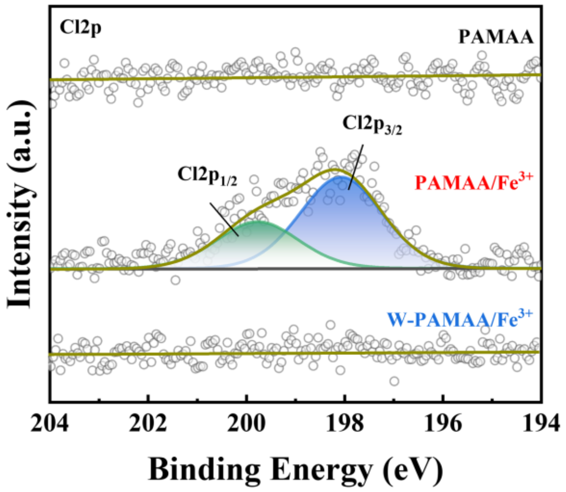

Fig. S10 The Cl2p spectrum of PAMAA, PAMAA/Fe^{3+}, and W-PAMAA/Fe^{3+} hydrogel fibers.

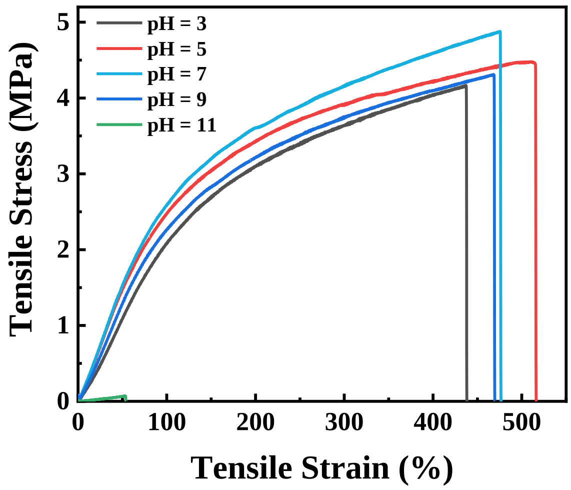

Fig. S11 Mechanical properties of W-PAMAA/Fe^{3+} hydrogel fibers after 120 days of swelling in an environment with a pH of 3-11.

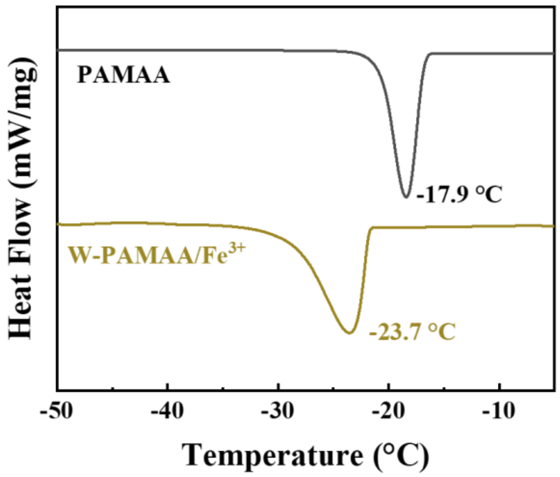

Fig. S12 DSC of PAMAA and W-PAMAA/Fe^{3+} hydrogel fibers.

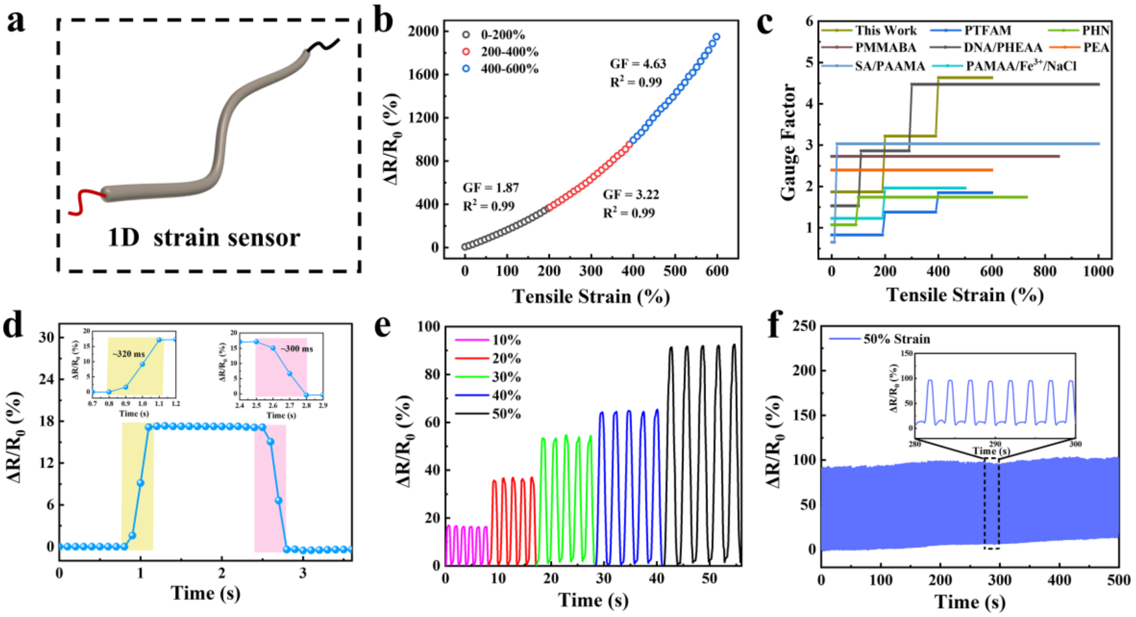

Fig. S13 (a) Schematic diagram of the 1D strain sensor based on the W-PAMAA/Fe^{3+} hydrogel fiber connected to electrodes. The red line was the positive pole and the black line was the negative pole. (b) the $\Delta R/R_0$ of the 1D strain sensor during stretching. (c) Comparison of the 1D strain sensors with other hydrogel-based sensors. (d) The response and recovery delay of the 1D strain sensor. (e) the $\Delta R/R_0$ of the 1D strain sensor at 10-50% tensile strain. (f) The long-cycle stability of 1D strain sensors.

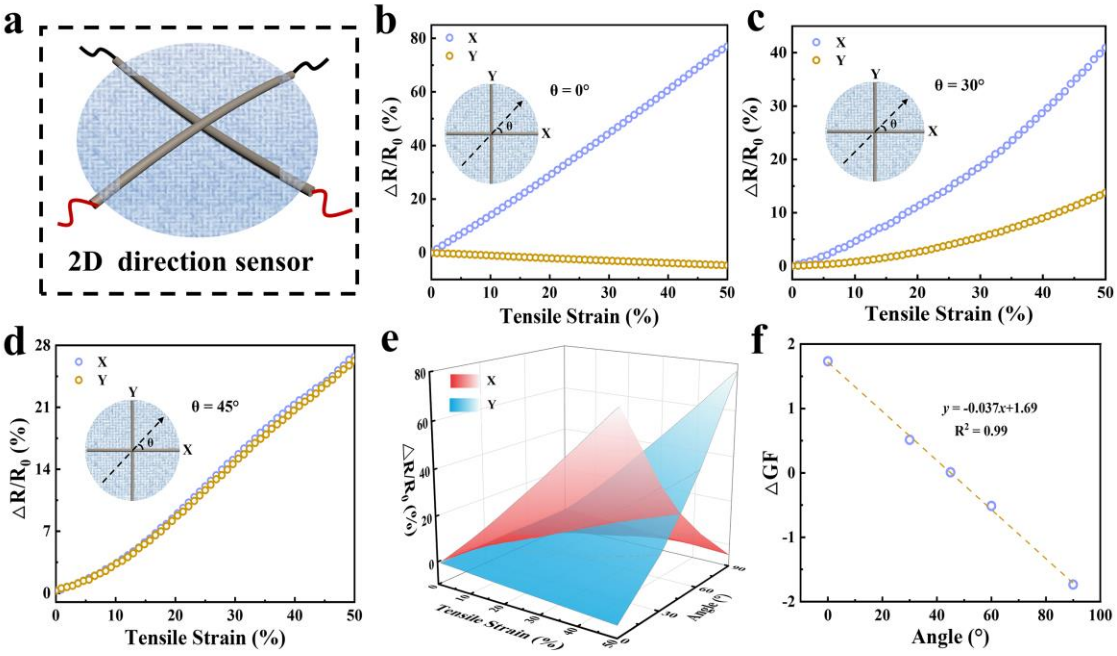

Fig. S14 (a) Schematic diagram of the 2D direction sensor based on the W-PAMAA/Fe^{3+} hydrogel fiber. The red line was the positive pole and the black line was the negative pole. The $\Delta R/R_0$ of two fibers when the 2D direction sensor was stretched at (b) 0°, (c) 30°, and (d) 45°. (e) The $\Delta R/R_0$ during the interaction of tensile angle and strain. (f) The directional selectivity factor of the 2D direction sensor.

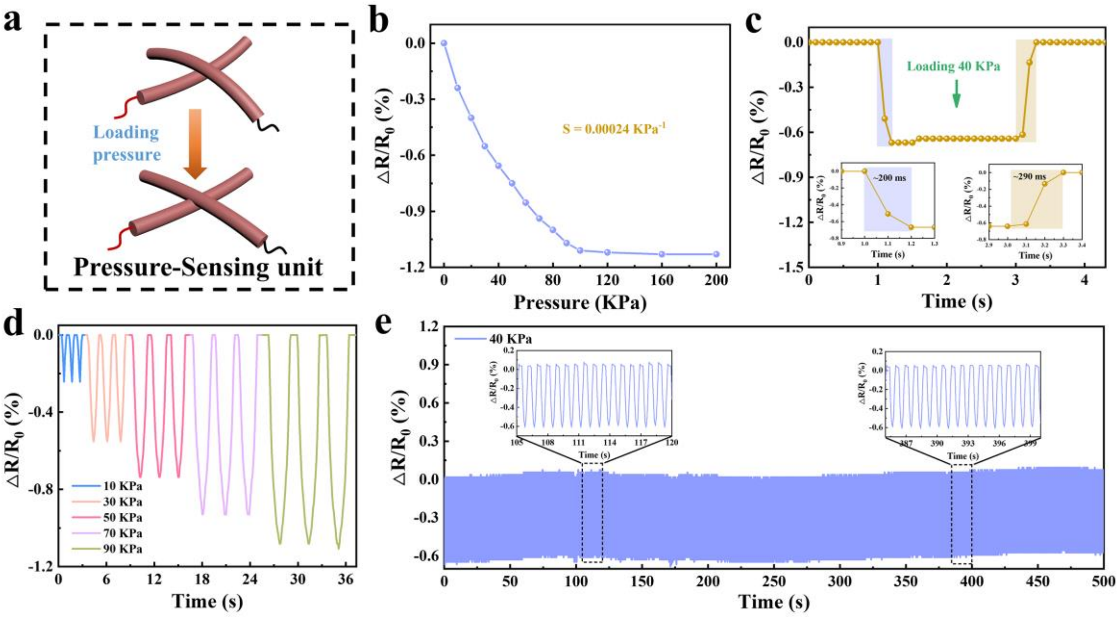

Fig. S15 (a) Schematic diagram of the pressure-sensing unit based on the W-PAMAA/Fe^{3+} hydrogel fiber. The red line was the positive pole and the black line was the negative pole. (b) the $\Delta R/R_0$ of the pressure-sensing unit during pressuring. (d) The response and recovery delay of the pressure-sensing unit. (e) the $\Delta R/R_0$ of the pressure-sensing unit at 10-90 KPa pressure. (f) The long-cycle stability of the pressure-sensing unit.
